# A Case Report of Giant Ascending Aortic Aneurysm: Role of Multimodality Imaging

**DOI:** 10.1055/s-0041-1730005

**Published:** 2021-10-12

**Authors:** Shabir H. Shah, Muhammad A. Shah, Abudar A. M. Alganadi, Halia Z. Alshehri, Mudasir S. Mir, Abdulaziz M. Alshammari

**Affiliations:** 1 Department of Adult Cardiac Surgery, King Fahad Medical City, Riyadh, Saudi Arabia; 2 Department of Adult Cardiology, King Fahad Medical City, Riyadh, Saudi Arabia

**Keywords:** giant ascending aortic aneurysm, hemiarch replacement surgery, aortic valve replacement surgery, multimodality imaging

## Abstract

Giant ascending aortic aneurysm (AscAA >10 cm) is an uncommon entity with a variable presentation. The size of the aneurysm, rapid expansion, and calcification are associated with an increased risk of rupture. Atherosclerosis is the most common etiology of aortic aneurysm in the elderly population. Multimodality imaging can be wisely used for diagnosis, risk stratification, and follow-up. We herein report a case of successfully repaired giant calcified AscAA with a maximum diameter of 10 cm. We also provide a brief discussion on the role of multimodality imaging.

## Introduction

Giant ascending aortic aneurysm (AscAA) is a rare disease with an asymptomatic course initially. Dissection or rupture of the aneurysm is the most dreadful complication. 1 The risk of rupture is closely related to the diameter of the aneurysm, increasing substantially at diameters greater than 6 cm. 2 Conservative management leads to low life expectancy. Surgical management is the only treatment option in such cases. Surgical repair of AscAA carries high mortality if combined with aortic valve replacement or concomitant arch replacement. The size of the aneurysm, rapid expansion, and calcification are associated with an increased risk of rupture. 3 We herein report a case of successfully repaired giant calcified AscAA with a maximum diameter of 10 cm.

## Case Presentation



**Video 1** Transthoracic echocardiography showing short axis at aortic valve level with tricuspid valve and central no coaptation. 2D, two-dimensional; PRE, preoperative.


**Video 2** Transthoracic echocardiography showing giant aortic aneurysm in long axis view. 2D, two-dimensional; PRE, preoperative.


**Video 3** Aortic angiography showing large calcified ascending aortic aneurysm.


**Video 4** Postoperative echocardiography showing a normally functioning aortic prosthesis. POST, postoperative.


**Fig. 1:**
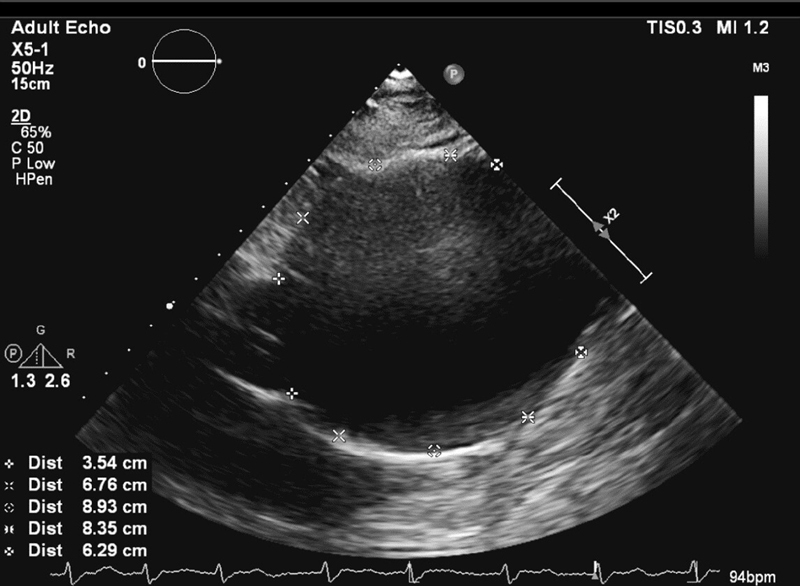
Transthoracic echocardiography showing a giant aortic aneurysm.

**Fig. 2:**
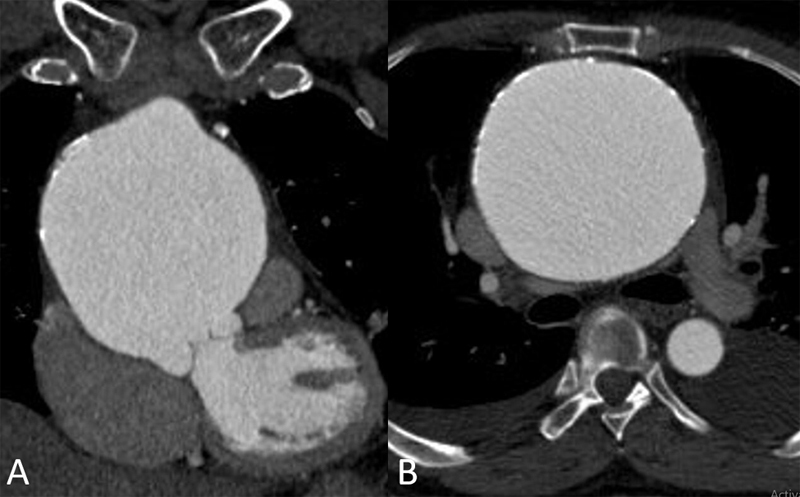
Cardiac computed tomography scan with contrast showing giant ascending aortic aneurysm in coronal ( **A** ) and axial plane ( **B** ) with evidence of compression of superior vena cava, pulmonary trunk, and right pulmonary artery.

**Fig. 3:**
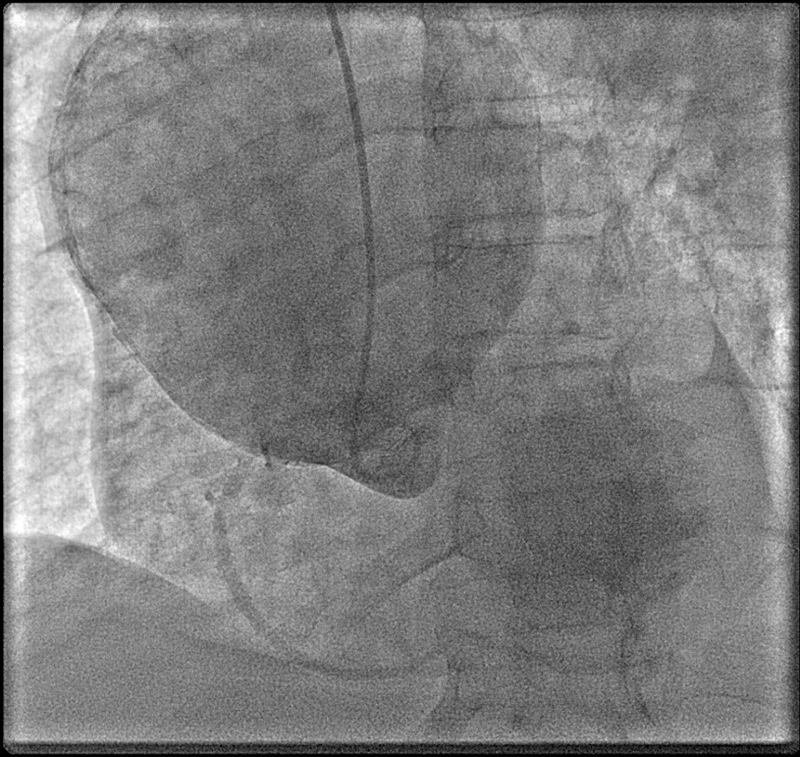
Coronary angiogram showing giant calcified ascending aortic aneurysm.

A 73-year-old male presented to our emergency department with acute decompensated heart failure. He suffered hypertension for almost 15 years and atrial fibrillation for 7 years. Electrocardiogram showed atrial fibrillation with tachycardia. Chest X-ray showed mediastinal widening along with mild congestion of both lungs and mild bilateral pleural effusions. Echocardiography showed a severely dilated ascending aorta (9 cm) with normal aortic root (the sinus of Valsalva = 3.7 cm × 3.8 cm × 3.5 cm and sinotubular junction = 3.5 cm; Fig. 1 ; Video 1 [available in the online version]). There was moderate-to-severe degenerative aortic regurgitation, and the ejection fraction was calculated as 50% ( Video 2 ; available in the online version). Computed tomography (CT) angiography showed giant calcified AscAA (measuring 10 cm × 9.7 cm) without evidence of dissection. The aneurysm was causing compression and narrowing of the superior vena cava, main pulmonary trunk, and right pulmonary artery ( Figs. 2 and 5 ). Coronary angiogram was performed due to risk factors of coronary disease and inability to assess coronaries on CT scan due to atrial fibrillation. It showed normal coronaries and the large calcified AscAA ( Fig. 3 ; Video 3 ; available in the online version).

**Fig. 4:**
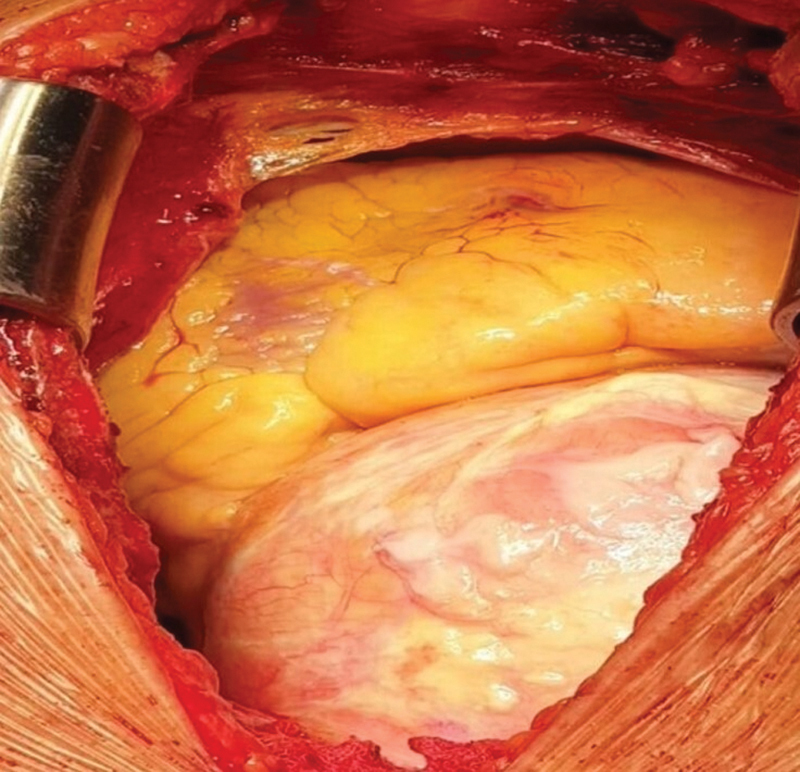
Giant aneurysm can be seen with spotty calcification after sternotomy.

**Fig. 5:**
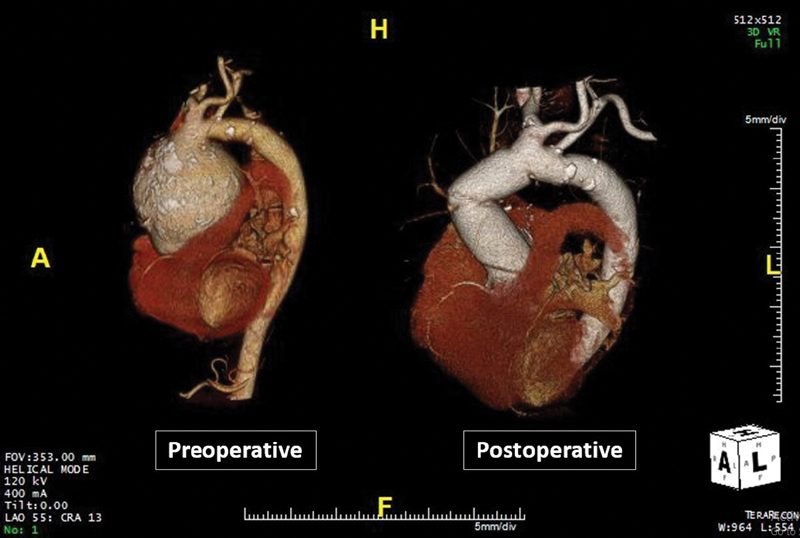
Three-dimensional volume-rendered images of cardiac computed tomography scan showing giant ascending aortic aneurysm preoperatively and aortic graft postoperatively.

After initial stabilization, the patient underwent successful replacement of the giant AscAA and hemiarch replacement with a vascular graft (size 32, Vascutek) and aortic valve replacement with a Magna Ease tissue valve (21 mm) under deep hypothermic circulatory arrest with antegrade cerebral perfusion through the right axillary artery ( Fig. 4 ). The overall postoperative course was uneventful except for high bilateral serous pleural drainage which settled spontaneously over the next 10 days. Biopsy of the resected ascending aorta showed fibroconnective tissue with myxoid degeneration and calcification. On follow-up, the patient was asymptomatic with a normally functioning aortic prosthesis on echocardiography ( Video 4 ; available in the online version). Follow-up cardiac CT scan revealed a well-seated vascular graft ( Fig. 5 ).

## Discussion

Although not defined in guidelines, giant AscAA is a term used for an aneurysm larger than 10 cm in its maximum diameter. 4 The ascending aorta is the most common site (60%) of thoracic aortic aneurysms. Giant aortic aneurysms are not commonly seen, as current guidelines recommend surgical correction when the diameter is more than 5.5 cm, as the risk of rupture significantly increases at reaching a diameter of 6 cm. 5 Zafar et al 6 recently proposed aortic height index (aortic size/height) for better prediction of risk of complications in AscAA rather than aortic size alone. The risk of complications was higher after 50 mm, suggesting earlier intervention to improve outcomes. The most common etiology is atherosclerosis in old age, while others include Marfan's syndrome, bicuspid aortic valve disease, giant cell arteritis, medial agenesis, and giant cell arteritis. AscAA usually follows an asymptomatic course and is mostly diagnosed incidentally on imaging. However, giant aneurysms can cause symptoms due to compression of neighboring structures like cardiac chambers, esophagus, trachea, and pulmonary artery. 7

Surgical repair is the only recommended treatment option for giant AscAA, although it is associated with significant complications such as aortic injury during sternotomy, bleeding, and end-organ ischemia. 7 8 Our patient had a giant AscAA and he presented with acute decompensated heart failure due to significant aortic regurgitation. The aneurysm was compressing superior vena cava, main pulmonary trunk, and right pulmonary artery. Successful aortic root–sparing surgical repair was performed, including replacement of the aortic valve.

In conclusion, giant AscAAs are often asymptomatic but can present with variable symptoms. Multimodality imaging is ideal for precise diagnosis and treatment. Surgical treatment is the standard approach with satisfactory outcomes.
